# Preparation of Graphene Oxide-Maghemite-Chitosan Composites for the Adsorption of Europium Ions from Aqueous Solutions

**DOI:** 10.3390/molecules27228035

**Published:** 2022-11-19

**Authors:** Galina Lujanienė, Raman Novikau, Edith Flora Joel, Karolina Karalevičiūtė, Sergej Šemčuk, Kęstutis Mažeika, Martynas Talaikis, Vidas Pakštas, Saulius Tumėnas, Jonas Mažeika, Kęstutis Jokšas

**Affiliations:** 1 SRI Center for Physical Sciences and Technology, Savanorių Str. 231, LT-02300 Vilnius, Lithuania; 2 SRI Nature Research Centre, Akademijos Str. 2, LT-08412 Vilnius, Lithuania

**Keywords:** Eu(III), adsorption, composite, graphene oxide, maghemite, chitosan, ANFIS

## Abstract

The adsorption of Eu(III) on composites synthesised from graphene oxide (GO), maghemite (MGH), and chitosan (CS) has been studied using different approaches. The physicochemical and morphological characteristics of the composites GO-MGH, GO-CS, GO-MGH-CS I, II, and III were determined by XRD, Mössbauer spectroscopy, FTIR, Raman spectroscopy, and TEM. According to the results of batch experiments, the maximum experimental adsorption capacity was 52, 54, 25, 103, and 102 mg/g for GO-MGH, GO-CS, GO-MGH-CS I, II, and III, respectively. The data obtained are in better agreement with the Langmuir, pseudo-second-order, and pseudo-first-order models only for GO-MGH. Thus, the adsorption of Eu(III) on the composites was a favourable, monolayer, and occurred at homogeneous sites. The nature of adsorption is chemical and, in the case of GO-MGH, physical. Tests of the composites in natural waters showed a high removal efficiency for Eu(III), Pu(IV), and Am(III), ranging from 74 to 100%. The ANFIS model has quite good predictive ability, as shown by the values for R^2^, MSE, SSE, and ARE. The GO-MGH-CS composites with the high adsorption capacity could be promising candidates for the removal of Eu(III) and the pre-concentration of Pu(IV) and Am(III) from natural waters.

## 1. Introduction

The release of radionuclides into the environment is usually a public concern. However, the energy crisis has led many countries to return to the use of nuclear energy as an additional source of green energy. The use of nuclear energy leads to an increase in nuclear waste produced in power plants (NPPs), as well as the associated problems of disposal and the risk of radionuclides being released into the environment. The accidents at the Chernobyl and Fukushima Daiichi NPPs can serve as notable examples of such emissions. Actinides are among the most harmful radionuclides released into the environment and have negative effects on ecological systems and human health due to their toxicity, carcinogenicity, and long half-life [1,2,3,4,5]. Actinides need to be controlled not only because of their possible release into the environment, but also when used in laboratories, as their handling in experimental studies requires caution and some restrictions. Tracer studies, including analogue studies, can help save time and reagents and reduce the amount of toxic waste that needs to be specifically disposed of due to the high environmental contamination requirements. Available technologies for removing radionuclides from polluted waters and monitoring activity concentrations can protect the environment. There are several methods for radionuclide removal, such as solvent extraction, ion exchange, coagulation, electrodeposition, and reverse osmosis, while adsorption attracts more attention due to its high efficiency, simplicity, and low cost [6,7,8,9,10,11,12,13,14,15].

Nanotechnologies are widely used in medicine, cosmetics, and food production, as well as in waste management and in the restoration of areas affected by man-made disasters. However, advanced technologies with lower energy consumption and less labour are needed to control radiological situations. Currently, much attention is being paid to the use of environmentally friendly nanocomposites based on natural materials due to their non-toxicity and biodegradability. One of the most promising materials, chitosan (CS), and its numerous modifications, have been used for wastewater treatment to remove various pollutants, including radionuclides [16,17].

Currently, there is a great demand for low-cost nanomaterials with high adsorption capacity for use under harsh environmental conditions to remove radionuclides. Researchers are interested in nanomaterials and nanoparticles with a thickness of several atomic layers, such as graphene oxide (GO), polymer nanosheets, magnetite (MG), ferrites, maghemite (MGH), layered double hydroxides, etc., due to the many highly reactive regions at the edges and basal planes with increased specific surface area. They are expected to be used in various applications [18,19].

Graphene oxide is an oxidised derivative of graphene with a large number of oxygen-containing functional groups such as carboxyl (COOH), epoxy (C–O–C), carbonyl (C=O), and hydroxyl (OH) groups on its base planes and edges. It has high chemical stability and a large surface area. Graphene oxide has found wide application in various fields, for example, in the fabrication of electrochemical sensors for the determination of tetrabromobisphenol A [20], refractive index sensors [21], multi-mode surface plasmon resonance absorbers [22], and as an adsorbent for the removal of various types of organic and inorganic pollutants [23,24,25,26,27]. The presence of oxygen-containing functional groups makes it promising for the preparation of composites with high adsorption capacity [28]. Recently published data have shown that GO and its modifications are very effective for the adsorption of Am(III), Pu(IV), Eu(III), and other radionuclides from contaminated solutions [29,30,31,32].

The use of composites with magnetic nanoparticles (MNPs), maghemite, and ferrites controlled by an external magnetic field enables the development of new technologies to remove highly toxic radionuclides from the environment. Composites of graphene oxide with magnetic particles, such as maghemite and magnetite, showed the improved adsorption properties and the ability to be magnetically removed. However, a disadvantage of these composites is that GO-MGH/MG tends to fold and form laminated microstructures, whereas this problem can be solved by linking the oxygen-containing groups of GO and the amino groups of chitosan. Thus, the GO-MGH/MG-CS composites used to remove metals and dyes showed high adsorption capacity, biocompatibility, and biodegradability [10,33,34,35,36,37].

The adsorption of Eu(III) was studied on sodium dodecyl sulphate-modified molybdenum disulphide (83 mg/g) [1], magnetic amidoxime-functionalised MCM-41 (25 mg/g) [2], magnetite decorated graphene oxide (46 mg/g) [10], titanate nanorings (115 mg/g) [12], zeolite (24 mg/g) [38], and magnetite nanoparticles (20 mg/g) [39].

In addition, its adsorption on graphene oxide, maghemite, and chitosan, as well as on a chitosan-containing bioadsorbent (*Saccharomyces cerevisiae* immobilised in glutaraldehyde cross-linked chitosan (SCGLUTCS)) materials was investigated [40,41,42,43]. The research results showed that the adsorption capacity of GO, MGH, CS, and SCGLUTCS increased with increasing initial concentration of europium ions, while the maximum adsorption capacity was 28.7, 33.5, 114.9, and 19.4 mg/g, and the equilibrium state was reached in 30, 240, 60, and 60 min, respectively. Moreover, the adsorption behaviour of Eu(III) was pH-dependent for all adsorbents, and the adsorption capacity of GO changed insignificantly with increasing ionic strength, while it decreased for MGH. Europium removal efficiency increased as a function of sorbent mass in the range of 10–500 mg, reaching a maximum at 100 and 150–200 mg for CS and SCGLUTCS, respectively, while the adsorption capacity of these materials decreased. The adsorption of Eu(III) on GO, MG and SKGLUTH had a spontaneous endothermic character, and increasing the temperature in the range of 20–60 °C had a positive effect on the removal of europium ions. Although europium has been studied on various adsorbents, we have not found any publications on the adsorption of europium on GO-MGH/MG-CS composites, while GO-MG-CS composites have been used for the removal of hexavalent chromium [35] and organic dyes [36] and showed excellent adsorption parameters.

The adaptive neuro-fuzzy inference system (ANFIS) is a multi-layered network that emerged from a combination of fuzzy logic and artificial neural network approaches and is used to model non-linear changes in complex systems and enables work with small data sets. Furthermore, ANFIS has proven to be a powerful modelling tool in adsorption studies and has high predictive accuracy compared to response surface methodology, artificial neural networks, and general neural regression network [44,45,46,47]. The choice of the output variables for the adsorption modelling is very important, as the quality of the result depends on it. The best prediction is achieved when using intensive variables, such as adsorption capacity, comparison to extensive variables, equilibrium concentrations, or removal efficiency [48].

The main objective of this work was to prepare graphene oxide-maghemite-chitosan composites (for ecological applications, monitoring, and tracer studies) using different synthesis approaches, to characterise the composites, to study the adsorption of Eu (a chemical analogue of trivalent actinides [12]) on them in bath experiments, and to investigate the adsorption processes using kinetic models and adsorption isotherms. Furthermore, this study aimed to investigate the adsorption efficiency of the resulting composites with respect to Eu(III), Pu(IV), and Am(III) in natural waters and to apply ANFIS to predict the adsorption capacity of the composites as a function of initial concentration, pH, and contact time.

## 2. Results and Discussion

### 2.1. Material Characterisation

**Figure 1:**
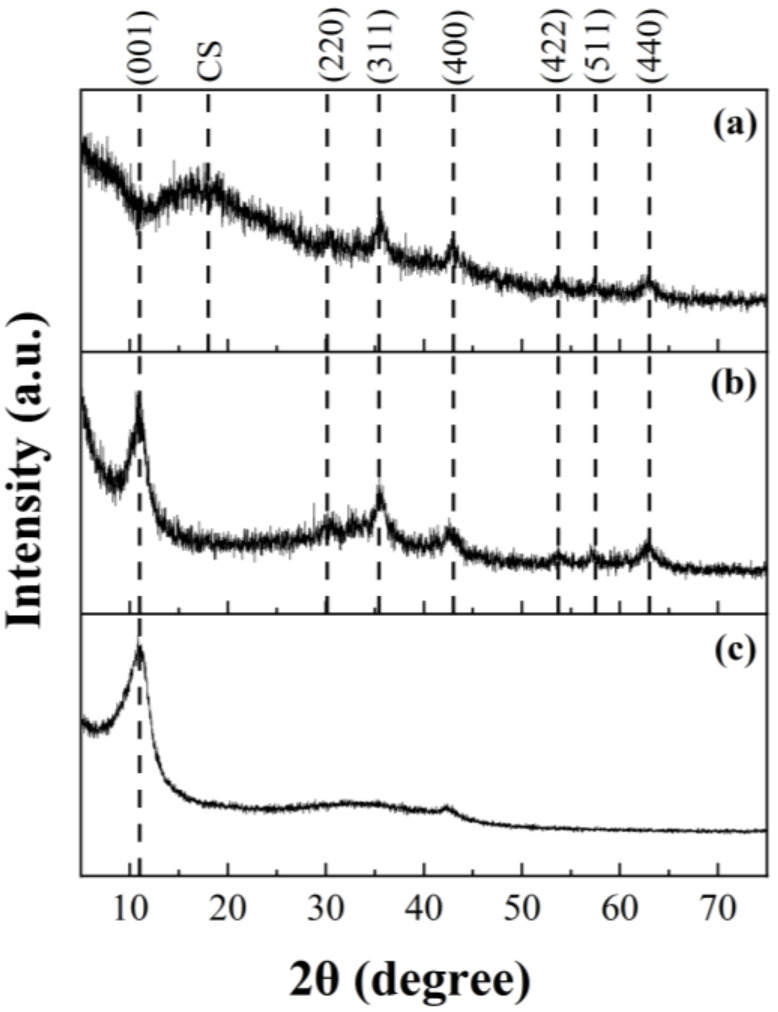
XRD pattern of GO-MGH-CS (**a**), GO-MGH (**b**), and GO (**c**).

Figure 1 shows the XRD patterns of GO, GO-MGH, and the composite of GO-MGH-CS performed in a 2θ range of 5–75°. In the XRD pattern of GO and GO-MGH, the diffraction peak at 10.98° corresponds to the typical (001) crystal plane of GO, which is also consistent with data published by other researchers [49,50]. The absence of a characteristic GO peak in the GO-MGH-CS composite can be explained by the possible overlap of the maghemite and chitosan peaks [51]. The values of 2θ = 30.2°, 35.4°, 42.5°, 53.7°, 57.5°, and 63.0°, found in GO-MGH and GO-MGH-CS, correspond to the crystallographic planes (220), (311), (400), (422), (511), and (440) of maghemite (*γ*-Fe_2_O_3_), respectively, which is consistent with the card (ICDD # 00-039-1346) and the literature data [52,53]. In addition, an amorphous peak at 2θ = 17.5° was found in the XRD pattern of the GO-MGH-CS composite, which can be attributed to chitosan [54].

**Figure 2:**
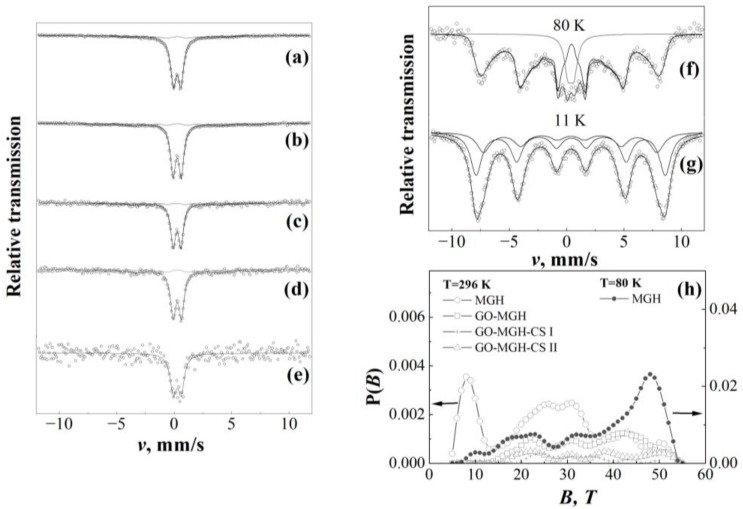
Mössbauer spectra of samples at 296 K: MGH (**a**), GO-MGH (**b**), GO-MGH-CS I (**c**), GO-MGH-CS II (**d**), GO-MGH-CS III (**e**), MGH at 80 K (**f**), and 11 K (**g**). The applied hyperfine field distributions are shown in (**h**).

Möessbauer spectroscopy data (Table 1 and Figure 2) show the presence of Fe^3+^ and absence of Fe^2+^ cations in MGH, GO-MGH, and GO-MGH-CS (I, II, III), indicating a maghemite phase rather than magnetite in the samples. Despite the similarity of their structures and magnetic properties, they differ in the presence of iron(III) cations in the case of maghemite and a mixture of iron(II) and iron(III) cations in the case of magnetite [53].

The spectra of samples MGH, GO-MGH, and GO-MGH-CS composites show a doublet in the centrum of the spectra (Figure 2a–e), which is due to small particles below 10 nm. However, in addition to the doublet, 18–19% of the spectra belong to the broad part described by the hyperfine distribution P(*B*), which can be explained by the particle size distribution and the presence of larger particles with a size ≥10 nm (Table 1). The average hyperfine field of distributions 25–35 *T* is 30–50% lower than that of bulk maghemite (*B_0_* = 49.1; 50.1 *T*) at room temperature [55]. The reduction of the hyperfine field can be explained by the collective excitations of the magnetic moment of the small maghemite nanoparticles. It is noted that further increase in the rate of such excitations due to smaller particles or higher temperature leads to superparamagnetic relaxation (flipping of the direction of the magnetic moment), resulting in a further decrease in the splitting of the spectrum and the appearance of a doublet at its centrum. At lower temperatures, the rate of superparamagnetic relaxation and excitations decreases, and the doublet in the spectra transforms into a magnetically split spectra, as can be seen in Figure 2f,g. The largest particle size of ~11 nm associated with hyperfine field distributions at room temperature was determined according to Equation (1), taking into account the 30% decrease in hyperfine field *B* associated with the decrease in particle volume *V* [56]:

(1) $$B=B_{0}\left(1-\frac{kT}{2KV}\right)$$

where *k* is the Boltzmann constant, *T* is the temperature, and *K* is the magnetic anisotropy of the maghemite.

At 80 K, the decrease in temperature *T* results in 87% of the spectral area being attributed to the hyperfine field distribution, with the average hyperfine field being 17–19% lower than that of the bulk maghemite at 77 K [55]. According to Equation (1), the particle size is 8.5 nm, but there are also smaller particles that cause a doublet in the spectrum. The temperature dependence of these spectra is characteristic for nanoparticles of 6 nm size, as a comparison with previous data [57,58]. The percentage of maghemite mass *M* of the samples (Table 1) was calculated according to the ratio resulting from the assumption that the area of the spectrum is proportional to the amount of maghemite and the thickness of the sample mass. The samples had a mass thickness of 20–40 mg/cm^2^, with the exception of GO-MGH-CS III with a greater thickness of 110 mg/cm^2^.

**Figure 3:**
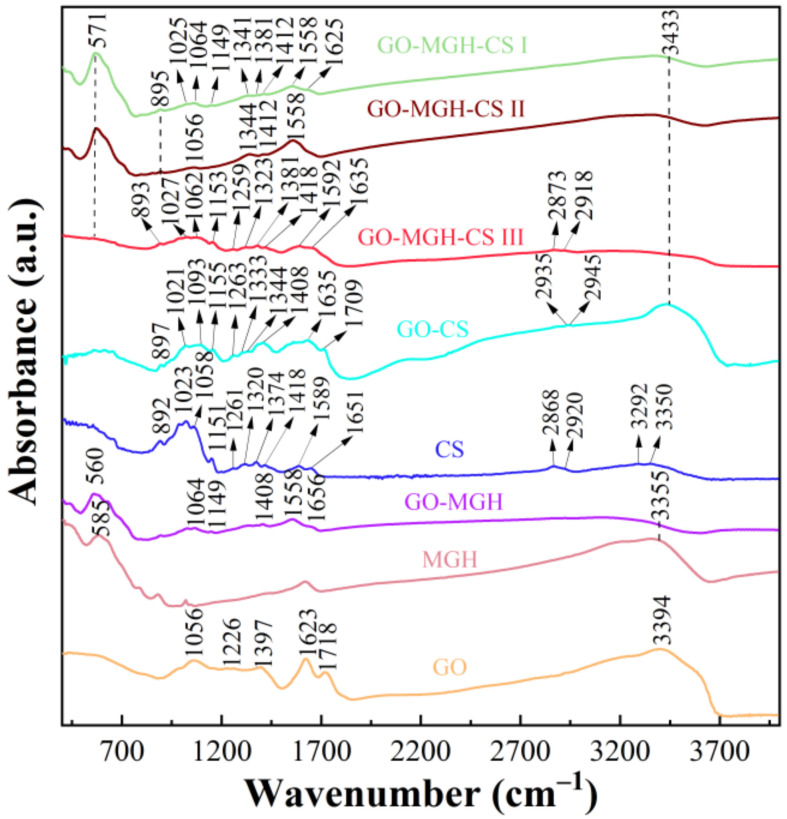
The FTIR spectrum of the original materials and composites.

The FTIR results provide information about the functional groups (GO, MGH, and CS) and their transformation in the composites GO-MGH, GO-CS, and GO-MGH-CS (I, II, III). In the spectrum of GO, bands at 1226, 1623, and 1056 cm^−1^ were found to be associated with C–O–C, aromatic C=C, and epoxy C–O stretching vibrations, respectively (Figure 3), while the bands at 1718 and 1397 cm^−1^ correspond to carboxyl C=O stretching vibrations [59]. A characteristic band of Fe–O stretching vibrations at 585 cm^−1^ was found in MGH [60]. Furthermore, the resulting maghemite has a well-ordered structure, as evidenced by broad bands in the 580 and 800 cm^−1^ regions [61]. The spectrum of GO-MGH contains bands corresponding to the functional groups of graphene oxide and maghemite. The position and intensity of these bands change. The band at 1226 cm^−1^ disappears and a new band forms at 1149 cm^−1^, indicating the interaction of graphene oxide with maghemite [62]. Moreover, the O–H stretching vibrations in the spectra of GO, MGH, and GO-MGH correspond to a broad band at about 3000–3700 cm^−1^. For CS, the band at 892 cm^−1^ corresponds to the CH bending of the monosaccharide ring. The bands at 1023 and 1058 cm^−1^ correspond to the C–O stretching. The signal at 1151 and 1261 cm^−1^ can be attributed to the asymmetric C–O–C stretching and the bending vibrations of the hydroxyls in the chitosan. The presence of C–N of the amide III and C=O of the amide I of the residual N-acetyl groups is confirmed by bands at 1320 and 1651 cm^−1^, respectively. However, the band at about 1550 cm^−1^ corresponding to the N–H of the amide II was not found because it is overlapped by other bands. The signal at 1374 and 1418 cm^−1^ is associated with symmetrical deformations of CH_3_ and bending of CH_2_. The band at 1589 cm^−1^ corresponds to the N–H bending of the primary amine. The presence of asymmetric and symmetric C–H stretching is confirmed by the bands at 2868 and 2920 cm^−1^, respectively. The absorption bands at 3292 and 3350 cm^−1^ correspond to the N–H and O–H stretching vibrations, respectively [63]. In the spectra of GO-CS and GO-MGH-CS (I, II, III), the absorption bands of graphene oxide and chitosan are combined, and in the case of GO-MGH-CS composites, the absorption bands of maghemite. Compared to the spectra of the original materials, a number of changes in the intensity and shift of the bands can be observed in the spectra of the composites. The band associated with N–H stretching also disappears in the spectra of these composites. For the composites GO-MGH-CS I, II, and III, the intensity of the bands associated with C–H stretching of chitosan and Fe–O maghemite, respectively, decreased significantly. Based on the obtained and published data [64,65], it can be assumed that the interaction is caused by the formation of covalent bonds between the oxygen-containing functional groups of GO/GO-MGH and CS.

**Figure 4:**
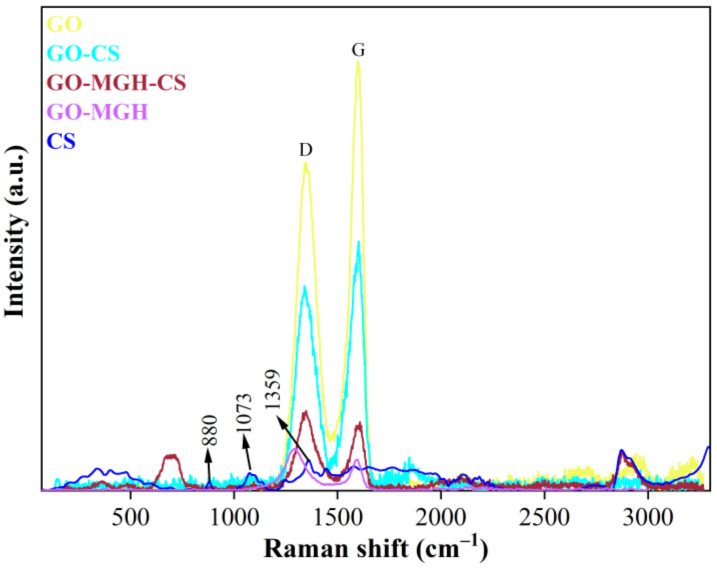
The Raman spectrum of GO, GO-CS, GO-MGH-CS, GO-MGH, and CS.

Raman spectroscopy showed a band at 880 cm^−1^ in the spectrum of chitosan belonging to NH_2_ (Figure 4). The bands at 1073 and 1359 cm^−1^ can be attributed to the stretching vibrations of the glycosidic bond, the ether bond, and the methyl group, respectively [66]. The spectra of GO and composites containing GO show characteristic D and G bands in the ranges 1295–1347 cm^−1^ and 1596–1609 cm^−1^. The D band is associated with structural defects originating from the oxygen-containing functional groups in the basal plane of the disordered carbon, and the G band characterises the stretching vibrations of the carbon atoms upon sp^2^ hybridisation [67]. Furthermore, a large intensity ratio of the D and G bands (*I_D_*/*I_G_*) indicates a decrease in the oxygen-containing groups in GO [68]. The resulting *I_D_*/*I_G_* values for the composites were higher than those of the original GO (0.76) and were 0.82 (GO-CS), 1.16 (GO-MGH-CS), and 1.36 (GO-MGH), which could be due to the involvement of oxygen-containing groups in the interaction with the functional groups of CS and MGH.

**Figure 5:**
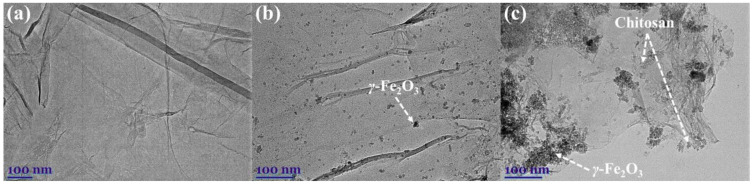
The TEM images of GO (**a**), GO-MGH (**b**), and GO-MGH-CS (**c**).

TEM images show the morphology of graphene oxide, GO-MGH, and GO-MGH-CS (Figure 5). It can be seen that GO (Figure 5a) has a sheet structure with curved edges, on the surface of which mainly single maghemite particles (with a rare case of aggregates visible in the image) with a size of <10 nm are uniformly distributed in the GO-MGH composite (Figure 5b). In the GO-MGH-CS composite (Figure 5c), the GO sheets are agminated by chitosan as a bridge between the layers [64], and the maghemite particles are aggregated on the surface of GO.

### 2.2. Adsorption Studies

#### 2.2.1. Effect of the Initial Concentration of Eu(III) and the Adsorption Isotherms

It has been shown (Figure S1a,d in the Supplementary Materials) that with an increase in the initial Eu(III) concentration, the adsorption capacity of the composites increases and saturation reaches 200 mg/L for GO-MGH, GO-CS, and GO-MGH-CS I. For GO-MGH-CS II and GO-MGH-CS III, saturation had not yet been reached, and the adsorption capacity increased in direct proportion to the increase in concentration, indicating a large number of active sites. The maximum adsorption capacity/efficiency for GO-MGH, GO-CS, GO-MGH-CS I, II, and III were 52/99, 54/99, 25/99, 103/99, and 102/100 (mg/g)/(%), respectively. It was also found that the weight percentage of chitosan in the composites GO-MGH-CS II and III had no effect on the adsorption capacity. The lower adsorption capacity of GO-MGH-CS I is apparently due to the cross-linking of graphene oxide and chitosan by glutaraldehyde, which makes some active sites of GO inaccessible to Eu(III) [69].

**Figure 6:**
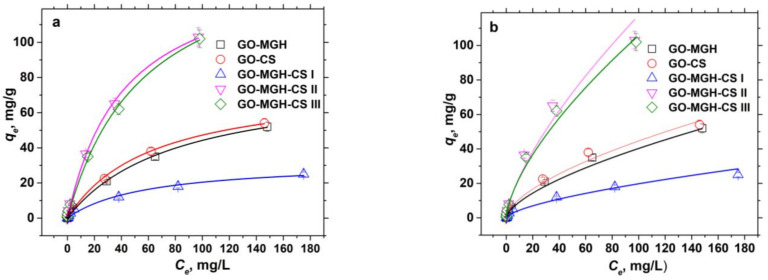
The non-linear adsorption isotherms of the Langmuir (**a**) and Freundlich (**b**).

The obtained data were fitted with the non-linear isotherms of Langmuir (Equation (2)) and Freundlich (Equation (3)). The results of the fitting are shown in Figure 6a,b and in Table 2. The Langmuir and Freundlich isotherms are the most widely used models [10,64,70] and suggest, in the case of Langmuir, a uniform distribution of active sites with equal adsorbate affinity on a homogeneous adsorbent surface where monolayer adsorption takes place after which the adsorbates no longer interact with each other, while in the case of Freundlich, multilayer adsorption takes place on a heterogeneous surface with interactions between the adsorbates.

(2) $$\;\;\;\;\;\;\;q_{e}=K_{L}q_{m}c_{e}/\left(1+K_{L}c_{e}\right)$$

(3) $$q_{e}=K_{F}c_{e}^{1/n}$$

where *K_L_* (L/mg) is the Langmuir constant and *K_F_* [(mg/g)(L/mg)] is the Freundlich constant; *q_m_* (mg/g) is the theoretical maximum of *q_e_*; *1/n* is the Freundlich intensity parameter.

The Langmuir model describes the adsorption of europium ions on composites better than the Freundlich model, as shown by the obtained values of the *R^2^*, and the values of the maximum adsorption capacity are close to the values of the equilibrium adsorption capacity *q_e_*. Moreover, the adsorption of Eu(III) was favourable on all composites, as indicated by the values of the separation factor (*R_L_*) (Equation (4)), since the adsorption is favourable at 0 < *R_L_* < 1 and unfavourable at *R_L_* > 1 [70].

(4) $$R_{L}=1/\left(1+K_{L}C_{0}\right)$$

Based on the suggestions of the Langmuir model, the adsorption of Eu(III) at the homogeneous sites of GO-MGH, GO-CS, and GO-MGH-CS (I, II, III) thus proceeded according to a monolayer type of adsorption without interactions in the adsorbate-adsorbate system after adsorption. The comparison of the maximum adsorption capacity of the studied composites with published data on the removal of Eu(III) showed that the composites GO-MGH-CS II and III had the highest adsorption capacity compared to GO-MGH, GO-CS, GO-MGH-CS I, and the other adsorbents (Table 3), which could be due to a large number of active sites. Moreover, the *q_m_* value of the composite GO-MGH is higher than that of maghemite and graphene oxide used separately, as well as for a number of other adsorbents, except chitosan. On the other hand, the composite GO-CS is inferior to only chitosan and MnO_2_/graphene oxide in the adsorption parameters, while the composite GO-MGH-CS I is inferior to almost all adsorbents except magnetite and magnetic amidoxime-functionalised MCM-41.

#### 2.2.2. Effect of pH

One of the most important factors affecting adsorption is the pH due to variation of Eu(III) speciation because, at a pH of <5, Eu^3+^ dominates, while at a pH of >5, the hydrolysis species of Eu(OH)_2_^+^ and Eu(OH)^2+^ predominate [15,74]. The adsorption capacity and efficiency of the GO-MGH-CS composites (I, II, III) increased with increasing pH (Figure S1b,e), while ion adsorption was consistently high for GO-MGH-CS II and III at a pH of >4 and for GO-MGH-CS I at a pH of >6. Similar behaviour of europium ions was observed in studies with *Saccharomyces cerevisiae* immobilised in glutaraldehyde cross-linked chitosan and graphene oxide nanosheets [40,43]. The pH of the point of zero charge (pH_PZC_) for GO-MGH-CS composites is 7.5, which leads to electrostatic repulsion at a pH of <7.5 between europium ions and the positively charged surface of the composites. Therefore, the adsorption of ions in the range of 2–7.5 can be explained by a chemical mechanism, such as complexation [40,43]. However, at a pH of >7.5, the adsorption processes are due to the electrostatic attraction between europium ions and the deprotonated surface of the composites [10,69]. The adsorption of Eu^3+^ on GO-CS increases in the pH range of 2–6, possibly due to complexation mechanisms, and remains at a high level at pH 7 and 8 as result of electrostatic attraction. The adsorption capacity/efficiency increases sharply at a pH of >5 for the GO-MGH composite, probably due to electrostatic attraction, as the observed pH_PZC_ values are 3.9–4.2 for GO and 4.5–4.9 for MGH, which are close to the published data. 4.1–4.5 [23,40] and 4.8 [41] were observed, respectively. It can therefore be assumed that the composite GO-MGG has a negative surface charge at pH 5 and above, which is consistent with the adsorption data.

#### 2.2.3. Kinetic Studies

**Figure 7:**
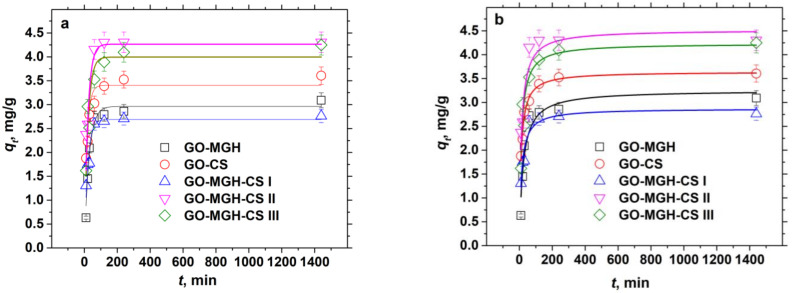
Fitting the pseudo-first-order kinetic model (**a**) and the pseudo-second-order kinetic model (**b**) to the experimental values.

Kinetic studies have shown (Figure S1c,f) that adsorption equilibrium is reached in 30 min for GO-MGH and GO-MGH-CS (I, II) composites and in 60 min for GO-CS and GO-MGH-CS III. This is faster compared to other similar adsorbents, e.g., the adsorption equilibrium of Eu(III) on magnetite decorated graphene oxide was reached in 240 min [10] and, on graphene oxide-chitosan, in 60 min [15], which is two times slower than on GO-MGH and GO-MGH-CS (I, II). In order to investigate the kinetics and the nature of adsorption of Eu(III) on the composites, the obtained data were fitted to the pseudo-first-order (5) and pseudo-second-order (6) nonlinear kinetic models [70].

(5) $$q_{t}=q_{e}\left(1-e^{-K_{1}t}\right)$$

(6) $$q_{t}=q_{e}^{2}K_{2}t/\left(1+K_{2}q_{e}t\right)$$

where *q_t_* (mg/g) is the amount of Eu(III) adsorbed per unit mass of composite (mg/g) at time *t* (min); *K_1_* (min^−1^) and *K_2_* [g/(mg·min^−1^)] are the rate constants for pseudo-first-order and pseudo-second-order, respectively. The results of the fitting with kinetic models and their parameters are shown in Figure 7 and Table 4, respectively.

The experimental data are in better agreement with the pseudo-second-order model, as shown by the values of *R^2^*, and the values of the maximum adsorption capacity were on average 1.4 times greater than the values of the equilibrium adsorption capacity *q_e_*. However, the adsorption of Eu(III) on GO-MGH is better described by a pseudo-first-order kinetic model. Thus, the pseudo-second-order model indicates the occurrence of chemisorption in the Eu(III)-GO-CS and Eu(III)-GO-MGH-CS (I, II, III) systems, while the pseudo-first-order indicates the physical nature of adsorption in the Eu(III)-GO-MGH system [75]. Furthermore, the physical adsorption of Eu on GO-MGH and its chemical adsorption on GO-MGH-CS (I, II, III) and GO-CS can be explained by studies on the influence of pH on adsorption. The kinetic studies were carried out at a pH of 5, at which the surface charge of the composites GO-MGH-CS (I, II, III) and GO-CS is positive, while GO-MGH is negative, leading to the predominance of chemical processes in the first case and physical processes in the second.

### 2.3. Adsorption Mechanism

**Figure 8:**
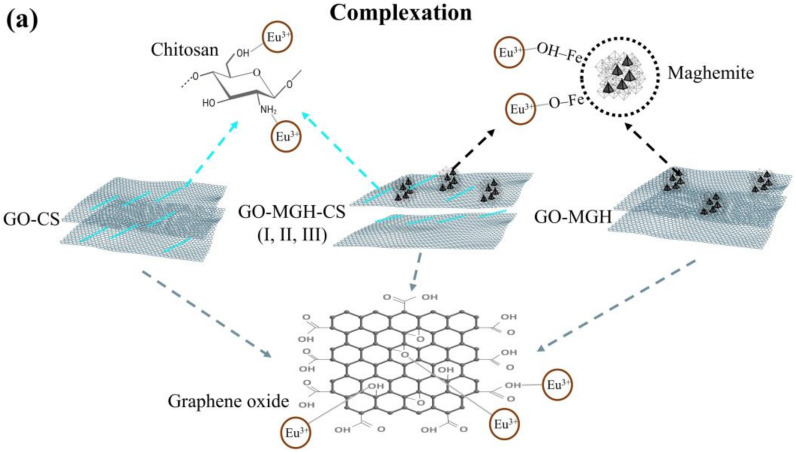

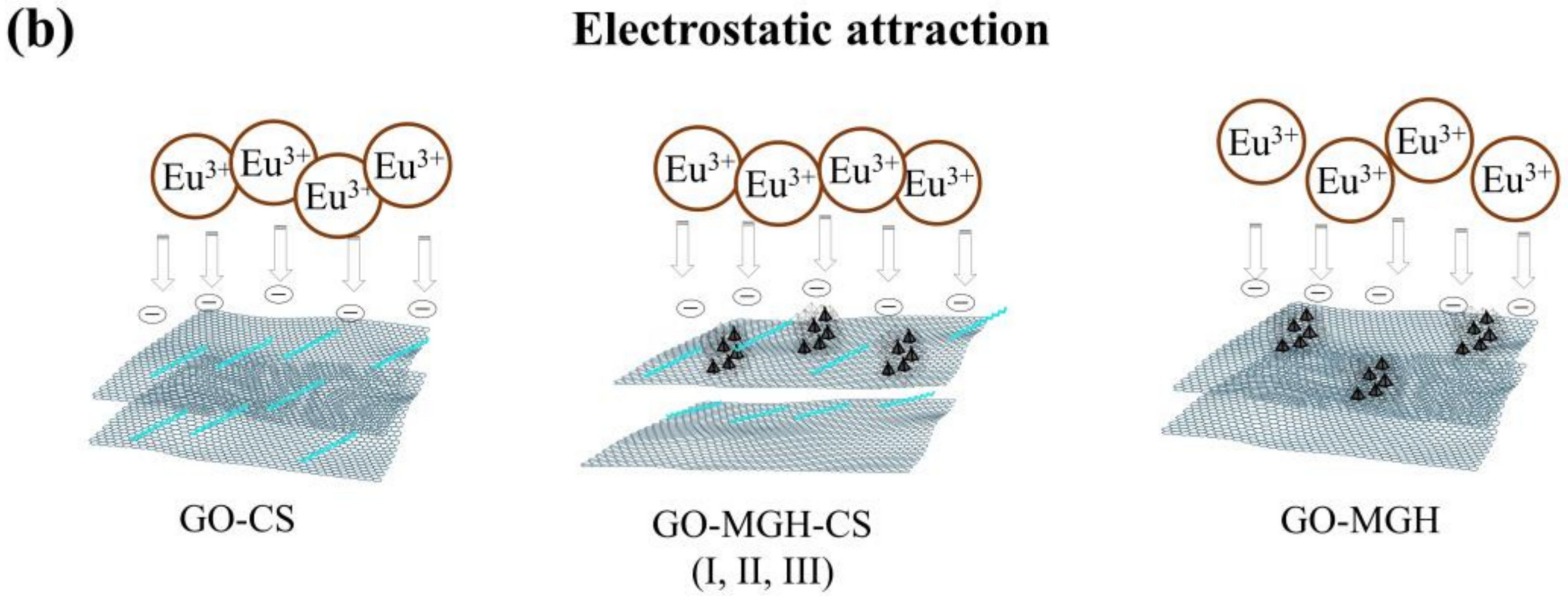
Proposed mechanism of adsorption of Eu(III) on composites by complexation (**a**) and electrostatic attraction (**b**).

The results of the study on the influence of pH and kinetics indicate the chemical and physical nature of the adsorption of europium ions on the composites. In studies [40,41,42,71,76] on the adsorption of europium ions on graphene oxide, MnO_2_/graphene oxide, chitosan, amidoxime-functionalised magnetic chitosan microparticles, maghemite using X-ray photoelectron spectroscopy, FTIR, and Eu(III) were found to interact with the following functional groups of graphene oxide: C=O (carbonyl and carboxyl groups), C–O (epoxy group), O–H, C=C, C–C (alkoxy group), chitosan (NH_2_ and OH), and maghemite (Fe–O/OH) and Fe(III)-*γ*-Fe_2_O_3_). The mechanism of adsorption on these materials involves complexation and electrostatic attraction, as shown by the data on the influence of pH and ionic strength on adsorption. Based on the experimental and published data, it can be assumed that europium adsorbs to composites due to complexation and electrostatic attraction (Figure 8).

### 2.4. Removal of Eu(III), Pu(IV), and Am(III) from Natural Waters

**Figure 9:**
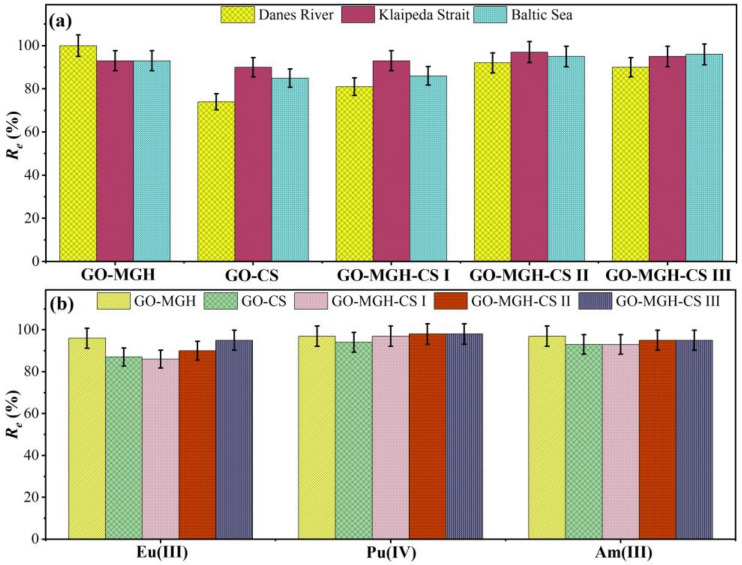
Removal of Eu(III) from different types of natural waters (**a**) and comparison of adsorption efficiency of the composites for Eu(III), Pu(IV), and Am(III) in seawater samples (**b**).

The data on the adsorption of europium by composites from the natural waters of the Danes River, the Klaipeda Strait, and the Baltic Sea are shown in Figure 9a and indicate that the adsorption efficiencies ranged from 74–100, 90–97, and 85–96%, respectively. The experiment was conducted under near-natural conditions (normally Eu concentration ranges between 1 mg/L in freshwater and 1.1 × 10^−6^ in seawater) without adding stable Eu to the water samples and using dialysis tubes with seven days of contact. The high efficiency of all sorbents studied was observed for Eu^3+^ and Eu(CO_3_)^+^ as the predominant species of Eu(III) in natural waters at pH 7.8 [74]. Data on the pre-concentration of Eu(III), Pu(IV), and Am(III) from seawater samples, when the composites were added to seawater samples for 24 h, also showed high efficiency for all composites studied (Figure 9b). These preliminary data suggest that the composites could be used for monitoring purposes as well as for long-term exposition and pre-concentration of actinides from environmental samples followed by radiochemical analysis and measurement by alpha or mass spectrometry.

### 2.5. Adaptive Neuro-Fuzzy Interference System

The ANFIS architecture (Figure S2) was developed based on MATLAB R2021 using Gaussian (Gausesmf2) with subtractive clustering (the range of influence of 0.5, squash factor of 1.25, acceptance rate of 0.5, rejection rate of 0.15). For training, we used 15 (GO-MGH, GO-CS, GO-MGH-CS I, II,) and 10 (GO-MGH-CS III) data, six data for testing, and six data for checking. The hybrid method based on least squares estimation and the back-propagation algorithm was used to optimise the modelling [44]. To reduce the influence of higher magnitude factors on the ANFIS architecture, the data were normalised by Equation (7) before entering the network and then trained with an error tolerance of zero and a number of training cycles (epochs) of 100 iterations. The ANFIS architecture thus consists of six layers. The first layer relates to the input data: pH, ion concentration, and contact time. The second is a fuzzy layer. The third is a product and normalised layer with four rules for GO-MGH and GO-MGH-CS (I, II), with two and five rules for GO-CS and GO-MGH-CS III, respectively. This is included from the fourth to the sixth layer—defuzzy, total output, and output (adsorption capacity). The performance of the network was estimated based on the fit of the normalised experimental and predicted data according to the mean squared error (MSE), the average relative error (ARE), and the sum of squared errors (SSE) calculated using Equation (8), Equation (9), and Equation (10), respectively:

(7) $${Ů}_{norm}=\frac{Ū-{Ů}_{min}}{{Ů}_{max}-{Ů}_{min}}$$

(8) $$MSE=\frac{1}{N}\sum_{i=1}^{N}{\left({Ῐ}_{i}-{Ῑ}_{i}\right)}^{2}$$

(9) $$ARE=\frac{100}{N}\sum_{i=1}^{N}\left|\frac{{Ῐ}_{i}-{Ῑ}_{i}}{{Ῑ}_{i}}\right|\%$$

(10) $$SSE=\sqrt{\frac{\sum_{i=1}^{N}{\left({Ῐ}_{i}-{Ῑ}_{i}\right)}^{2}}{N}}$$

where Ů*_norm_* is the normalised value; Ů*_min_* and Ů*_max_* are the minimum and maximum values, respectively; Ū is a vector of values; *N* is the number of experimental points; ${Ῐ}_{i}$ is the predicted output; and ${Ῑ}_{i}$ is the experimental output.

**Figure 10:**
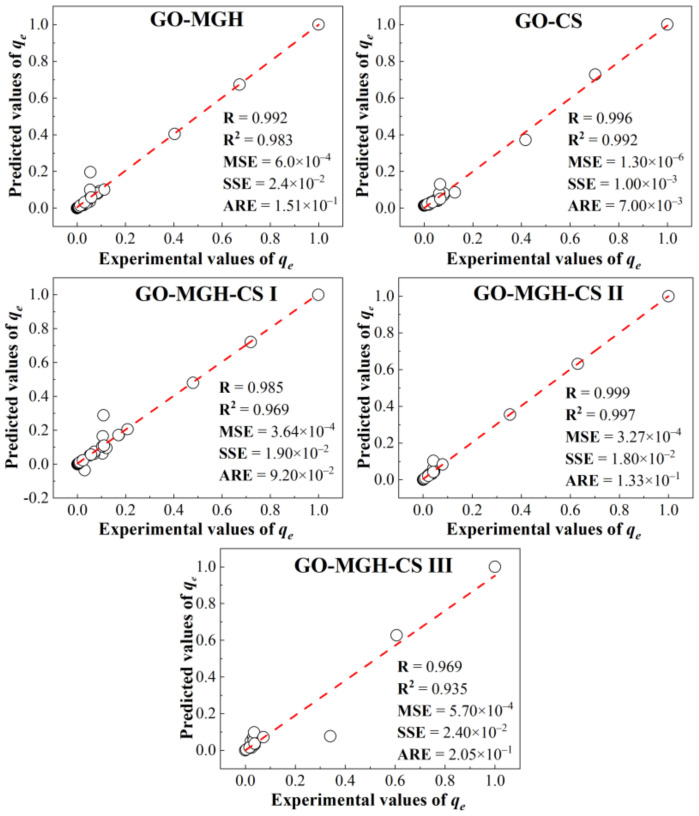
Comparison of the experimental data with the data predicted by ANFIS.

The results of the comparison between the experimental and predicted data are shown in Figure 10. There is good agreement between the predicted and experimental values, indicating high prediction accuracy. This is confirmed by the values of the correlation coefficient, which are close to one, and the values of MSE, SSE, and ARE, which are less than one. The results of the three-dimensional surface modelling (Figures S3–S5) show that the adsorption behaviour of Eu(III) on composites is complex and non-linear as a function of initial concentration, pH, and contact time. The ANFIS model is thus able to predict the adsorption capacity or behaviour with a fairly high accuracy, and its subsequent use can be useful in planning adsorption experiments.

## 3. Materials and Methods

### 3.1. Preparation of Composites

For the synthesis of GO, the Hummers method was applied with some modifications for GO using the graphite powder (<20 μm synthetic, Sigma-Aldrich, Switzerland) [77,78]. The graphite powder and NaNO_3_ were added to H_2_SO_4_, and then KMnO_4_ was added slowly and continuously. Hydrogen peroxide solution (30 wt%) was used to remove the excess of MnO_4_^−^ anions. The resulting mixture was centrifuged, and the precipitate was washed with Milli-Q water and alcohol and then dried at 70 °C under vacuum.

GO-CS composite was prepared by mixing solutions of graphene oxide and chitosan. Then, 3 g of chitosan was dissolved in 100 mL of 1% acetic acid solution, and 0.5 g of GO was dispersed in 100 mL of Milli-Q water under sonication and at room temperature. Then, the two prepared solutions were mixed and sonicated for 10 min to achieve a homogeneous solution. Then, the GO-CS was precipitated with 1 M NaOH in ethanol solution. Finally, the prepared composite was washed several times with C_2_H_5_OH (Absolute, Merck, Rahway, NJ, USA), C_2_H_3_N, and Milli-Q water until the pH was neutral. Before the GO-CS was used for adsorption experiments, it was dried at 50 °C for 24 h.

The synthesis of glutaraldehyde cross-linked GO-MGH-CS I with a weight ratio of GO:MGH:CS of 2.5:2.5:1 was carried out using hydrothermally aged MGH-NPs. For the synthesis of these MGH-NPs, we used concentrated solutions of FeCl_2_ and FeCl_3_ in a molar ratio of 1.1:2. The black mixture formed after the addition of the freshly prepared NaOH solution was stirred for several minutes and diluted with water. The mixture was washed several times with water, acidified to a pH of ~2, and washed again with water to obtain a slurry. After hydrothermal ageing in an ultrasonic bath at 80 °C for 30 min, the resulting suspension was dialyzed against 0.001 mol/L HCl. The resulting suspension was stored in a refrigerator at 4 °C.

Aqueous suspensions of MGH-NPs and graphene oxide were used to prepare GO-MGH composites. The suspensions were sonicated for 30 sec before being rapidly mixed in a ratio GO:MGH of 1 to 1 with a final MGH concentration of 5 g/L. The resulting suspension with a pH of ~5 to 6 was stored in the refrigerator for further synthesis.

The obtained GO-MGH suspension was mixed with CS (90–310 kDa, 75–85% deacetylated, Sigma Aldrich, Taufkirchen, Germany) solution (50 mg chitosan was dissolved in 25 mL, 2% (*v*/*v*) acetic acid solution) in the ratio GO-MGH to CS of 2:1. Then, 2 mL of 2% glutaraldehyde was added to the mixture, and the pH was adjusted to pH 9–10 with 1 mol/L NaOH solution. The resulting mixture was kept at 60 °C for 2 h with mechanical stirring. The composite was separated with a magnet, washed with water to neutral pH, and mixed three times with absolute ethanol. Finally, the resulting GO-MGH-CS I composite was dried at 50 °C for 16 h under vacuum.

For the synthesis of GO-MGH-CS II with a weight ratio of GO:MGH:CS of 2.5:2.5:1, MGH-NPs were prepared by adding 25% NH_4_OH to a solution of FeCl_2_ and FeCl_3_ (molar ratio 1.5:2) at 40 °C with stirring. The mixture solution was kept in the pH range of 9–10 and stirred vigorously at 60 °C for 1 h and then cooled to room temperature. The precipitate was separated with a magnet and washed several times with water. The obtained MGH-NPs were dispersed in water and added dropwise to GO aqueous solution (1 mg/mL). The mixture was mechanically stirred at 60 °C for 1 h. Then the GO-MGH composite was collected with a magnet and washed three times with water.

The GO-MGH-CS II composite was prepared by adding the CS solution (50 mg chitosan was dissolved in 25 mL, 2% (*v*/*v*) acetic acid solution) to the GO-MGH composite suspension. The pH of the mixture was adjusted to pH 9–10 with 1 mol/L NaOH solution and stirred at 60 °C for 2 h. The GO-MGH-CS II composite was collected with a magnet, washed with water to pH of ~7 and three times with absolute ethanol. The resulting composite was dried overnight in a vacuum at 50 °C.

The preparation of the composite GO-MGH-CS III was similar to that of the composite GO-MGH-CS II, with the weight ratio GO:MGH:CS remaining at 1:1:1.

### 3.2. Characterisation

The prepared composites were characterized using the D8 X-ray diffractometer (Bruker AXS, Bremen, Germany) for X-ray diffraction analyses, the Vertex 70v (Bruker Inc., Bremen, Germany) vacuum spectrometer with a spectral resolution of 2 cm^−1^ (using a KBr pellet for sample preparation) for Fourier transform infrared spectrometry, and the RAM II system (Bruker Inc., Bremen, Germany) (180° backscattering configuration) with a 1064 nm laser for recording Raman spectra. The morphology and particle size of the synthesised composites were investigated using transmission electron microscopy with a Tecnai G2 F20 X-TWIN (FEI, Eindhoven, Netherlands), resolution 0.25–0.102 nm. Mössbauer spectra were recorded at room temperature with the Mössbauer spectrometer (Wissenschaftliche Elektronik GmbH, Starnberg, Germany) in transmission geometry using the ^57^Co(Rh) source. Normos Dist software was used to analyse the spectra.

### 3.3. Batch Experiments

The adsorption experiments were performed with an initial Eu^3+^ concentration of 0.01–200 mg/L, a dosage of 1 g/L (ratio 1:1000 g/mL) of adsorbent, a temperature of 25 °C, and a pH range of 2 to 8. Initial pH values were monitored with a WTW inoLab Multi Level 1 m (Weilheim, Germany) and WTW pH electrode SenTix 41 (Weilheim, Germany) calibrated with standard buffers DIN 19266. The EuCl_3_ solutions were traced with ^152^Eu. Agitation speed was studied at 100, 200, and 300 rpm for 5 h and 24 h. The observed *q_e_* values varied within the range of uncertainties. For this reason, all experiments were conducted at 200 rpm. The adsorbent was separated after the desired time with a magnet or by centrifugation at 20,000× *g* for 15 min. Three sets of adsorption experiments were performed using the batch technique. Eu activity concentrations were measured by gamma spectrometry using HPGe detectors (GEM40P4-76, efficiency 42%, resolution 1.9 keV/1.33 Mev; and the coaxial well GWL-120-15-LB-AWT detector, resolution 2.25 keV/1.33 MeV). The details of the sorption experiment are described in previous publications [79,80].

The adsorption capacity and removal efficiency of GO-MGH, GO-CS, and GO-MGH-CS (I, II, III) composites were calculated by Equation (11) and Equation (12), respectively:

(11) $$q_{e}=\frac{\left(c_{0}-c_{e}\right)}{m}\times V\;\;$$

(12) $$R_{e}=\frac{\left(c_{0}-c_{e}\right)}{c_{0}}\times 100\%\;\;$$

where *q_e_* (mg/g) is the amount of Eu(III) adsorbed at equilibrium, *R_e_* (%) is the removal efficiency, *c*_0_ (mg/L) is the initial concentration of Eu(III), *c_e_* (mg/L) is the equilibrium concentration of Eu(III), *V* (L) is the working volume, and *m* (g) is the weight of the adsorbent.

### 3.4. Determination of the Point of Zero Charge

The pH of the point zero charge was measured using the pH drift method [81]. Test solutions of GO-MGH-CS (I, II, and III) composites (0.05–0.1 g) were equilibrated with 50 mL of 0.01–0.1 mol/L NaNO_3_ under argon flow and 25.0 ± 0.1 °C. The measured values were recorded 10 and 30 min after the addition of the titrant. The drifts of the measured potential were less than 2 mV between the readings after 10 and 30 min.

### 3.5. Removal of Europium, Americium, and Plutonium Ions from Natural Waters

The composites GO-CS, GO-MGH, and GO-MGH-CS (I, II, III) were tested for Eu adsorption from natural waters with a similar pH of 7.8 and varying salinity from 0 to 7 psu. Water samples from the Baltic Sea (55°44′53′′–21°02′05′′), the Danes River (55°42′40′′–21°08′10′′), and the Klaipeda Strait (55°43′49′′–21°04′34′′) were traced with ^152^Eu. The adsorbents were placed in dialysis tubes (cellulose membrane with a molecular weight cut-off of 14 kDa) and left in contact with the water samples for seven days under continuous stirring. The dialysis tubes were removed from the containers after seven days, and Eu activities in the water samples were measured by gamma spectrometry.

To test the suitability of the synthesised composites for pre-concentration of actinides from seawater, 1 L seawater samples were used. Pu(IV) and Am(III) were added to the water samples as a mixture of ^238^Pu, ^239^Pu, ^240^Pu, ^241^Pu, and ^241^Am. Then, the composites were added, and the samples were mixed for 1 h and left overnight. The composites were collected with a magnet or by centrifugation. The Am and Pu isotopes were measured by alpha spectrometry after radiochemical separation using the TEVA and TRU columns (Eichrom Industries). ^242^Pu and ^243^Am were used as tracers in the separation procedure. Further details can be found in previous publications [82,83,84].

## 4. Conclusions

In this study, the composites synthesised using different approaches have an agminated sheet structure, with the chitosan acting as a bridge between the layers, and the maghemite particles <10 nm in size and evenly distributed on the surface of the sheets. The results of the batch experiments show the dependence of the adsorption of europium ions on the composites on the initial concentration, pH, and contact time. The maximum experimental adsorption capacity of the composites was 52, 54, 25, 103, and 102 mg/g for GO-MGH, GO-CS, GO-MGH-CS I, II, and III, respectively, and was obtained at a pH of 5, a contact time of 1440 min, and an initial concentration of Eu(III) of 200 mg/L. Moreover, the composites GO-MGH-CS II and III showed the best result in terms of adsorption properties in batch experiments compared to the other composites. The Langmuir, pseudo-second-order, and pseudo-first-order models (for GO-MGH) better describe the adsorption data, indicating that the adsorption of Eu(III) occurs by monolayer, chemisorption, and on GO-MGH by physisorption. The composites remove Eu(III), Pu(IV), and Am(III) equally effectively from seawater samples, and the removal of Eu(III) from samples of different water types varied from 74 to 100%. The prediction results of ANFIS were close to the experimental data, and the three-dimensional surface modelling results show that the adsorption of europium ions on the composites was complex and non-linear. GO-MGH-CS composites have a high adsorption capacity, are easy to handle due to possible magnetic separation, and are environmentally friendly due to their weak antibacterial properties and biocompatibility. The preliminary results of this study indicate that the composites can be used for on-site radiation monitoring, long-term exposition, and pre-concentration of actinides from environmental samples including tracer studies associated with Am and Pu.

## Figures and Tables

**Table 1:** Parameters of Mössbauer spectra: *T*—measurement temperature, *M*—mass percentage of maghemite, *I_d_* and *I_B_*—relative area of doublet and hyperfine distribution or sextet, respectively, *δ*—isomer shift relatively to *α*-Fe, *Δ*(2ε)—quadrupole splitting (shift), *B*—hyperfine field.

Sample	*T,* K	*M, %*	Doublet			Hyperfine Field Distribution/Sextets			
			*I_d_*, %	*δ*, mm/s	*Δ*, mm/s	*I_B_*, %	*δ*, mm/s	*Δ*, mm/s	*B*, T
MGH	296	100	81	0.36 ± 0.01	0.66 ± 0.01	19	0.36 *	0 *	25.4 **
	80		13	0.48 ± 0.01	0.51 ± 0.04	87	0.46 ± 0.01	–0.14 ± 0.02	37.2 **
	11		0	-	-	61	0.49 ± 0.01	–0.07 ± 0.02	51.0 ± 0.2
						39	0.45 ± 0.01	–0.06 ± 0.03	46.8 ± 0.4
GO-MGH	296	32	81	0.36 ± 0.01	0.72 ± 0.01	19	0.36 *	0 *	34.6 **
GO-MGH-CS I	296	15	80	0.36 ± 0.01	0.68 ± 0.01	20	0.30 ± 0.06	0 *	29.9 **
GO-MGH-CS II	296	17	74	0.37 ± 0.01	0.73 ± 0.01	26	0.37 *	0 *	35.2 **
GO-MGH-CS III	296	0.6	100	0.37 ± 0.02	0.68 ± 0.03	-	-	-	-

Notes: * fixed value; ** average of distribution.

**Table 2:** The parameters of the isotherm models.

Models	Langmuir				Freundlich		
Parameters	*R^2^*	*q_m_,* mg/g	*K_L_*	*R_L_*	*R^2^*	*K_F_*	*1/n*
GO-MGH	0.998	80	0.013	0.278	0.659	2.097	0.642
GO-CS	0.995	77	0.016	0.238	0.540	2.681	0.611
GO-MGH-CS I	0.982	32	0.017	0.227	0.871	1.000	0.649
GO-MGH-CS II	0.999	147	0.024	0.172	0.967	4.794	0.695
GO-MGH-CS III	0.998	160	0.018	0.217	0.920	5.100	0.658

**Table 3:** Comparison of *q_m_* of different adsorbents for Eu(III).

Adsorbents	Conditions	*q_m_* (mg/g)	Refs.
Chitosan nanoparticles	pH = 3; T = 25 °C.	115	[42]
Maghemite	pH = 6; T = 25 °C.	34	[41]
Magnetite nanoparticles	pH = 5; T = 25 °C.	0.24	[39]
GO	pH = 5; T = 25 °C.	68	[71]
MnO_2_/graphene oxide		84	
Magnetic amidoxime-functionalised MCM-41	pH = 6; T = 25 °C.	27	[2]
Manganese dioxide@polypyrrole core/shell nanomaterial	pH = 6.5; T = 25 °C.	55	[72]
Carbonaceous nanofibers	pH = 4.5; T = 25 °C.	63	[73]
GO-MGH	pH = 5; T = 25 °C.	80	This work
GO-CS		77	
GO-MGH-CS I		32	
GO-MGH-CS II		147	
GO-MGH-CS III		160	

**Table 4:** The parameters of the kinetic models for Eu(III).

Models	Pseudo-First-Order				Pseudo-Second-Order		
Parameters	*R^2^*	*K_1_* (min^−1^)	*q_e Theo_* (mg/g)	*q_e Exp_* (mg/g)	*R^2^*	*K_2_* [g/(mg min^−1^)]	*q_e_* (mg/g)
GO-MGH	0.964	0.035	2.964	3.091	0.914	0.014	3.254
GO-CS	0.873	0.061	3.404	3.606	0.976	0.026	3.644
GO-MGH-CS I	0.904	0.050	2.689	2.763	0.931	0.027	2.872
GO-MGH-CS II	0.768	0.048	4.266	4.306	0.824	0.018	4.521
GO-MGH-CS III	0.851	0.048	3.995	4.249	0.914	0.016	4.320

## Data Availability

The data collected in this study are available on request from the corresponding author.

## Supplementary Materials

The following supporting information can be downloaded at: https://www.mdpi.com/article/10.3390/molecules27228035/s1, Figure S1: adsorption capacities and efficiencies of the composites as a function of initial concentration, pH, and contact time; Figure S2: ANFIS architecture; Figure S3: input-output surfaces of the ANFIS model for the GO-MGH and GO-CS composites; Figure S4: input-output surfaces of the ANFIS model for the GO-MGH-CS I and GO-MGH-CS II composites; Figure S5: input-output surfaces of the ANFIS model for the GO-MGH-CS III composite.
