# Supplementary material for: Preparation of Graphene Oxide-Maghemite-Chitosan Composites for the Adsorption of Europium Ions from Aqueous Solutions
Source: Molecules. 2022 Nov 19;27(22):8035. doi: 10.3390/molecules27228035 (PMC9694936; doi:10.3390/molecules27228035)
Supplement: Supplementary file 1 [file molecules-27-08035-s001.zip › molecules-2031203-supplementary.pdf]

Supplementary information

Preparation of Graphene Oxide-Maghemite-Chitosan Composites for the Adsorption of Europium Ions from Aqueous Solutions

Galina Lujanienė [1,*], Raman Novikau [1], Edith Flora Joel [1], Karolina Karalevičiūtė [1], Sergej Šemčuk [1], Kęstutis Mažeika [1], Martynas Talaikis [1], Vidas Pakštas [1], Saulius Tumėnas [1], Jonas Mažeika [2] and Kęstutis Jokšas [2]

[1] SRI Center for Physical Sciences and Technology, Savanorių Str. 231, LT-02300 Vilnius, Lithuania
[2] SRI Nature Research Centre, Akademijos Str. 2, LT-08412 Vilnius, Lithuania
* Correspondence: galina.lujaniene@ftmc.lt

Contents

Figure S1. Adsorption capacities and efficiencies of the composites as a function of initial concentration, pH, and contact time 2
Figure S2. ANFIS architecture 3
Figure S3. Input-output surfaces of the ANFIS model for the GO-MGH and GO-CS composites 4
Figure S4. Input-output surfaces of the ANFIS model for the GO-MGH-CS I and GO-MGH-CS II composites 5
Figure S5. Input-output surfaces of the ANFIS model for the GO-MGH-CS III composite 6

Figure S1.

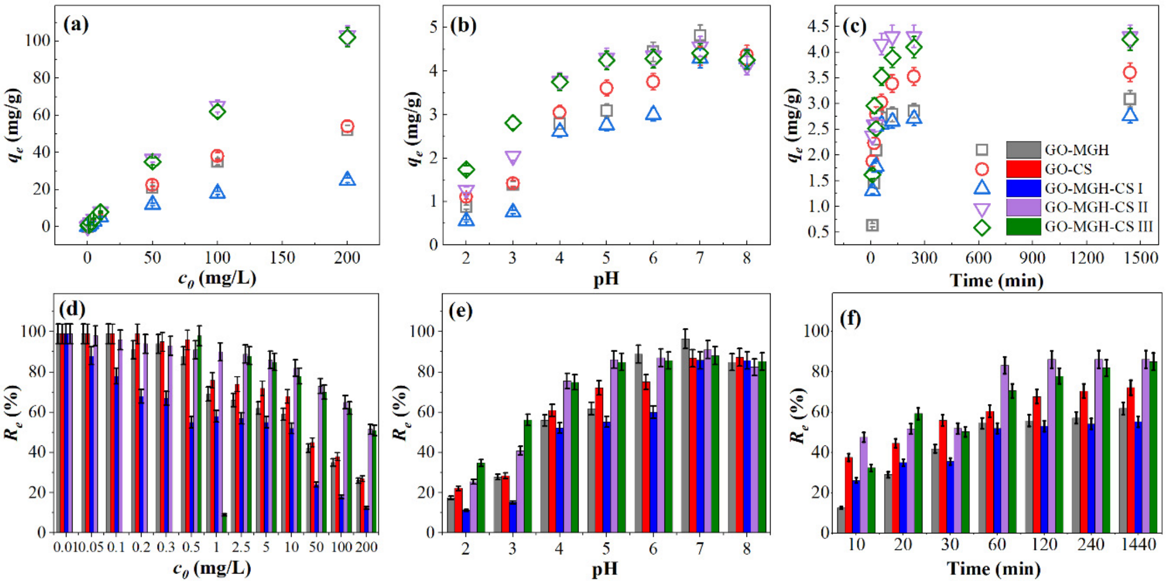

Figure S1. Adsorption capacities and efficiencies of the composites as a function of initial concentration (**a**, **d**), pH (**b**, **e**), and contact time (**c**, **f**).

Figure S2.

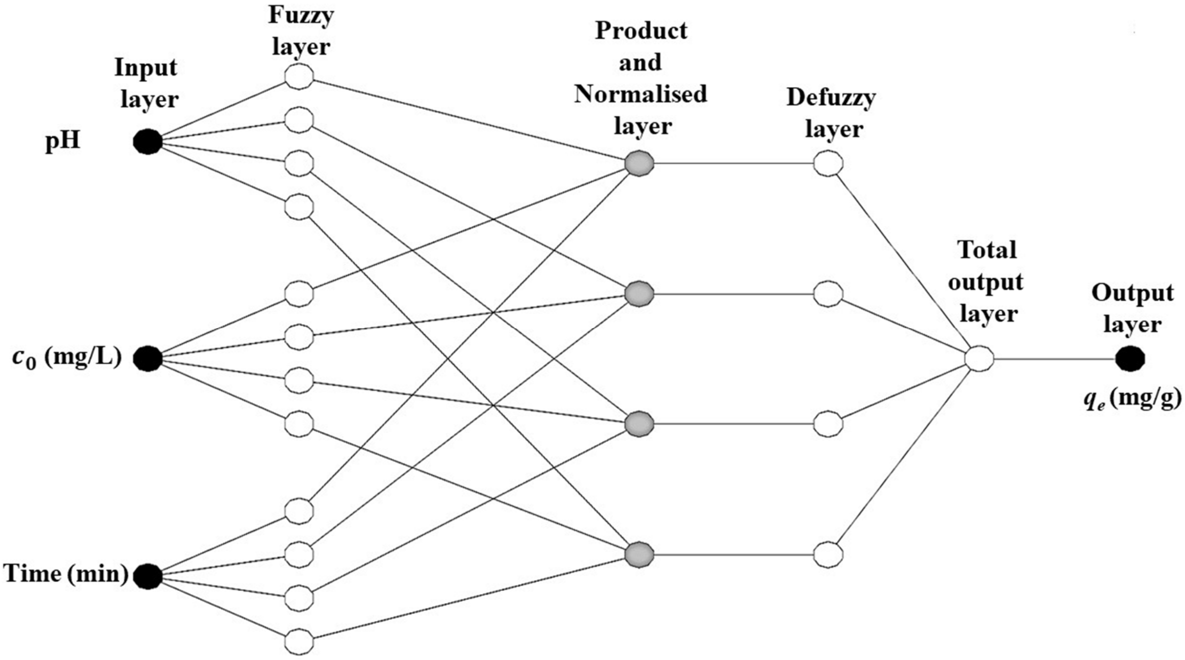

Figure S2. ANFIS architecture.

Figure S3.

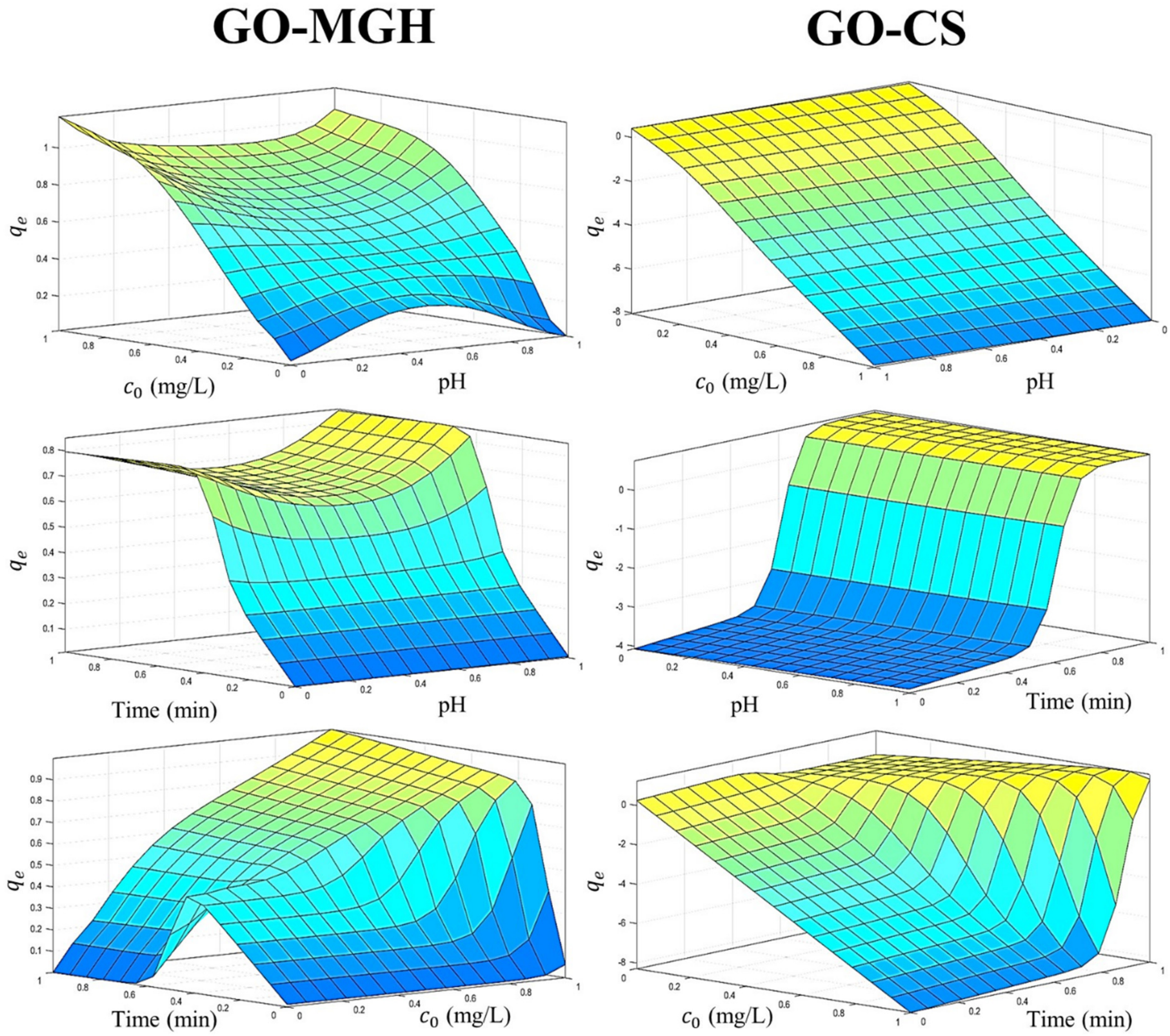

Figure S3. Input-output surfaces of the ANFIS model for the GO-MGH and GO-CS composites.

Figure S4.

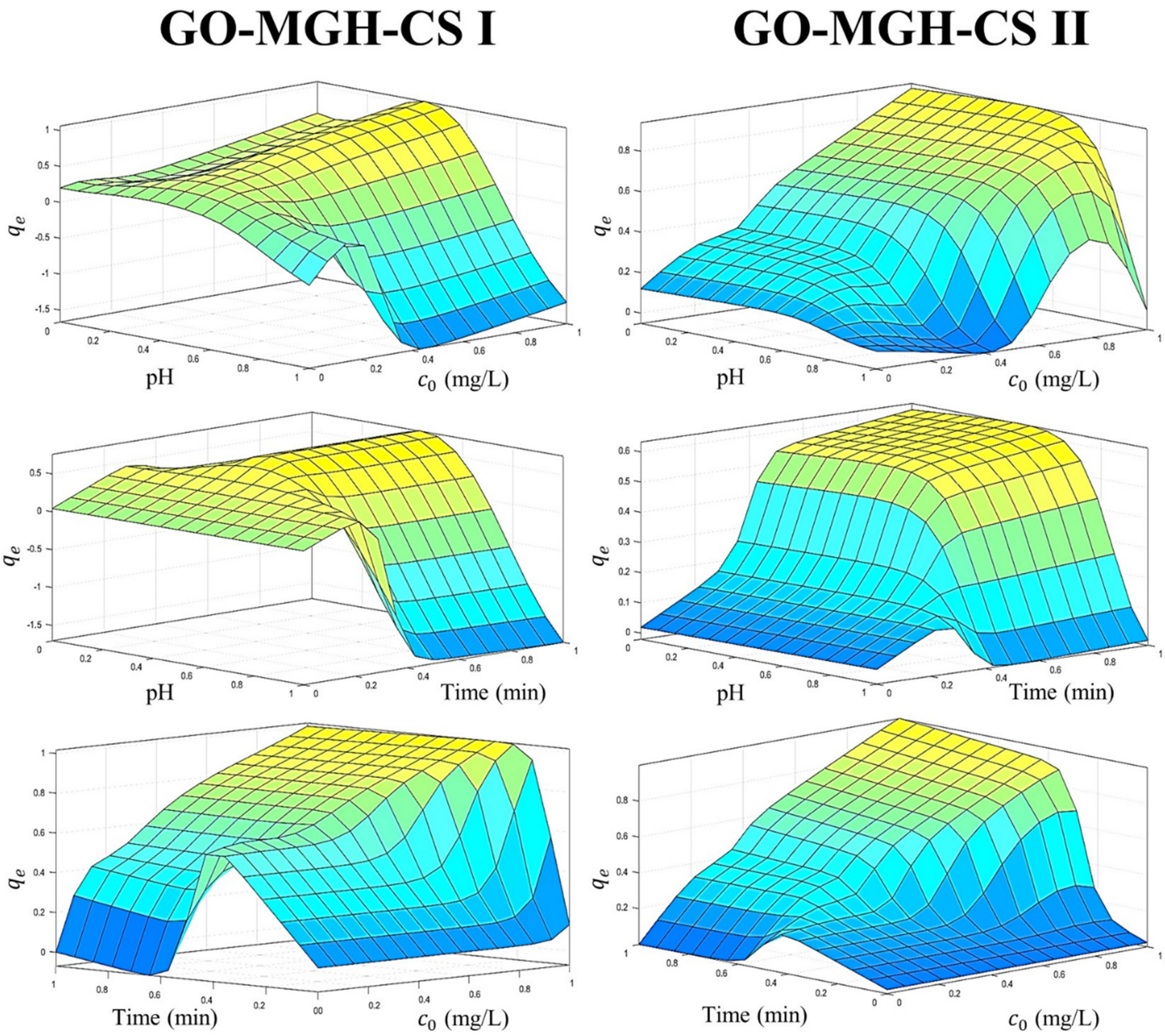

Figure S4. Input-output surfaces of the ANFIS model for the GO-MGH-CS I and GO-MGH-CS II composites.

Figure S5.

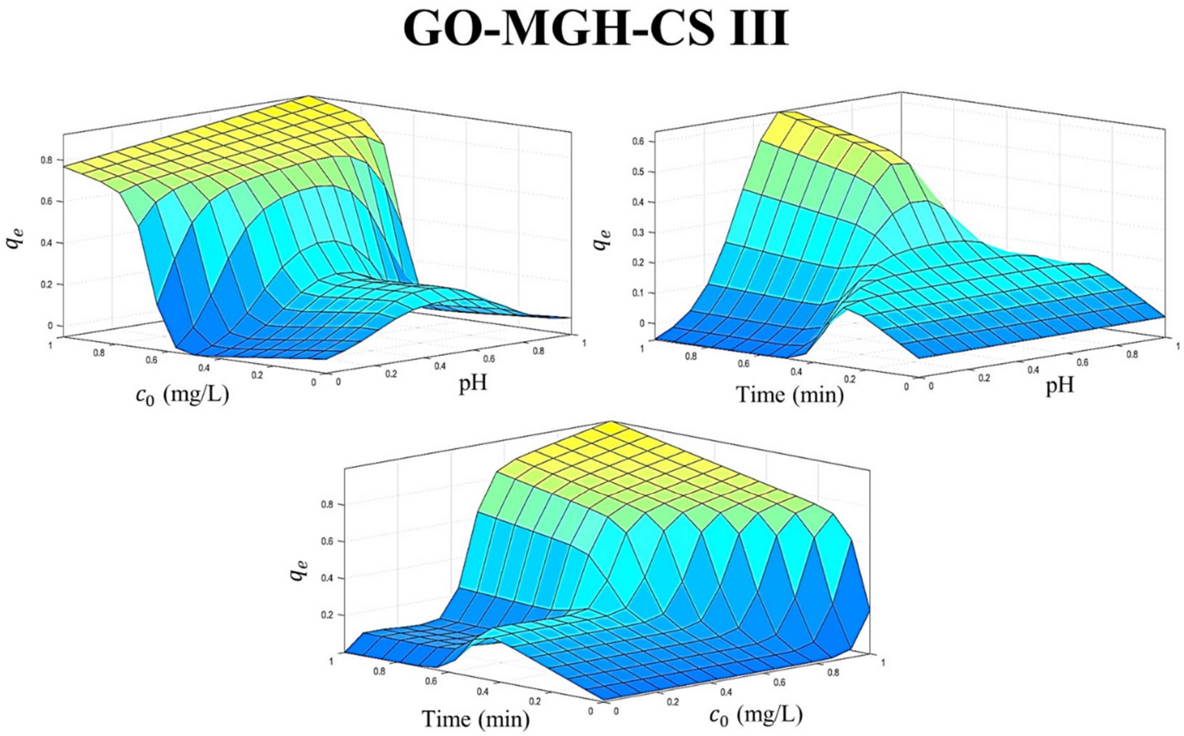

Figure S5. Input-output surfaces of the ANFIS model for the GO-MGH-CS III composite.
